# LC-MS Quantification of Site-Specific Phosphorylation Degree by Stable-Isotope Dimethyl Labeling Coupled with Phosphatase Dephosphorylation

**DOI:** 10.3390/molecules25225316

**Published:** 2020-11-14

**Authors:** Sin-Hong Chen, Ya-Chi Lin, Ming-Kuei Shih, Li-Fei Wang, Shyh-Shyan Liu, Jue-Liang Hsu

**Affiliations:** 1 Department of Veterinary Medicine, National Pingtung University of Science and Technology, Pingtung 91201, Taiwan; p10116004@mail.npust.edu.tw; 2 Department of Biological Science and Technology, National Pingtung University of Science and Technology, Pingtung 91201, Taiwan; linyachiheart@gmail.com; 3 Graduate Institute of Food Culture and Innovation, National Kaohsiung University of Hospitality and Tourism, Kaohsiung 81271, Taiwan; mkshih@mail.nkuht.edu.tw; 4 Hospitality and Tourism Research Center, National Kaohsiung University of Hospitality and Tourism, Kaohsiung 81271, Taiwan; lifeiso@mail.nkuht.edu.tw; 5 Research Center for Tropic Agriculture, National Pingtung University of Science and Technology, Pingtung 91201, Taiwan; 6 Research Center for Animal Biologics, National Pingtung University of Science and Technology, Pingtung 91201, Taiwan

**Keywords:** phosphorylation degree, stable-isotope dimethyl labeling, phosphatase dephosphorylation, LC-MS, SRM

## Abstract

Protein phosphorylation is a crucial post-translational modification that plays an important role in the regulation of cellular signaling processes. Site-specific quantitation of phosphorylation levels can help decipher the physiological functions of phosphorylation modifications under diverse physiological statuses. However, quantitative analysis of protein phosphorylation degrees is still a challenging task due to its dynamic nature and the lack of an internal standard simultaneously available for the samples differently prepared for various phosphorylation extents. In this study, stable-isotope dimethyl labeling coupled with phosphatase dephosphorylation (DM + deP) was tried to determine the site-specific degrees of phosphorylation in proteins. Firstly, quantitation accuracy of the (DM + deP) approach was confirmed using synthetic peptides of various simulated phosphorylation degrees. Afterwards, it was applied to evaluate the phosphorylation stoichiometry of milk caseins. The phosphorylation degree of Ser130 on α-S1-casein was also validated by absolute quantification with the corresponding synthetic phosphorylated and nonphosphorylated peptides under a selected reaction monitoring (SRM) mode. Moreover, this (DM + deP) method was used to detect the phosphorylation degree change of Ser82 on the Hsp27 protein of HepG2 cells caused by *tert*-butyl hydroperoxide (*t*-BHP) treatment. The results showed that the absolute phosphorylation degree obtained from the (DM + deP) approach was comparable with the relative quantitation resulting from stable-isotope dimethyl labeling coupled with TiO_2_ enrichment. This study suggested that the (DM + deP) approach is promising for absolute quantification of site-specific degrees of phosphorylation in proteins, and it may provide more convincing information than the relative quantification method.

## 1. Introduction

Protein phosphorylation is an important reversible post-translational modification that switches “on” or “off” signal transductions in a wide array of biological processes. Abnormal phosphorylation has been regarded as the cause of many human diseases [1,2,3]. To date, identification and site mapping of protein phosphorylation mainly relies on tandem mass spectrometry (MS/MS) in proteomic studies. However due to the low abundances of phosphoproteins compared to their unmodified counterparts and other non-phosphoproteins, only liquid chromatography associated with tandem mass spectrometry (LC-MS/MS) without proper sample pretreatment may not be sufficient for large-scale analyses of protein phosphorylation. In addition, the low compatibilities of phosphopeptides towards MS detection could also result in considerable errors [4]. Many methods targeted at enrichment of phosphorylated proteins/peptides have been developed to overcome these two limitations, and identification of protein phosphorylation has been significantly improved [5,6,7]. Nevertheless, quantitative analysis of the dynamic changes of phosphorylation in response to various stimulations, rather than merely identification, is expected to provide more valuable information underlying the physiological and pathological processes. Through years of studies, the approach that integrates stable-isotope labeling, selective enrichment, and LC-MS/MS has become the technical mainstream for relative quantification of protein phosphorylation [8,9]. For example, stable-isotope reductive methylation (also known as dimethyl labeling) coupled with various conventional enrichment methods, such as immobilized antibody [10], immobilized metal ion affinity chromatography (IMAC) [11], TiO_2_ particles [12], and strong cation-exchange chromatography (SCX) [13], has performed certain levels of feasibility. However, these methods for relative quantitation may not accurately reflect the real dynamic changes of phosphorylation degrees occurring in various physiological statuses, mainly because of the reversible and transient nature of phosphorylation and the lack of a common internal standard for normalizing the bias derived from the samples of different preparations. Thus, absolute quantification of the phosphorylation stoichiometry at a definite state is required for precisely deciphering the functions of phosphorylation modifications under various physiological conditions. For this purpose, Zhang et al. defined a “sample-splitting approach” by digesting the protein sample into peptides, which were then divided into two aliquots. After one aliquot was dephosphorylated with alkaline phosphatase, the N-terminuses of peptides in the dephosphorylated and phosphorylated aliquots were chemically modified with D_5_- and D_0_-propionyl groups, respectively. These two aliquots were then recombined, and the abundance ratio of D_5_/D_0_ peptide pairs was obtained by the matrix-assisted laser desorption/ionization (MALDI) measurement [14]. Owing to its simple workflow, the sample-splitting approach has been adopted by many studies. For example, coupled with methyl esterification (methanol-*d*_4_/methanol) of peptide C-termini, the sample-splitting method enabled Hegeman et al. to unambiguously identify the site-specific phosphorylation stoichiometries of two protein samples by comparing the chemically identical but isotopically distinct peptide species through µLC-MS analysis [15]. Even though there are limitations for the sample-splitting method, e.g., phosphorylation itself may cause differential cleavage of proteases, the hydrolyzed peptide mixtures may not be equally split, etc., the bias generated from sample splitting was generally considered marginal and acceptable [15,16].

However, more significant deviations usually result from the processing of chemical modification. For example, incomplete esterification or partial ester hydrolysis often occurs in acidic conditions, especially in methyl esterification of the carboxylic acid group of acidic amino acid residues (Asp/Glu), which may affect the efficiency of peptide sequencing and protein identification [17,18]. Additionally, some undesirable reactions, such as acylation on the side chain of Tyr residue, should be eliminated by extra operations (e.g., hydrolysis) prior to LC-MS analysis. In addition, propionylation of Lys residue may neutralize the positive charge of Lys side chain and diminish the sensitivity of peptide detection by mass spectrometry. Therefore, guanidination of the Lys side chain with *O*-methylisourea is required prior to dephosphorylation and propionylation treatments [14]. However, guanidination reaction is usually processed at an extremely alkaline condition (4 M barium hydroxide solution or NH_4_OH at pH 11) [19], which will lead to β-elimination of phosphopeptides and give an inaccurate quantification. To circumvent the side effects of chemical modifications, more efficient and milder stable-isotope labeling operations are quested by researchers.

In this study, stable-isotope dimethyl labeling (DM), a mild, efficient, and robust chemical modification [20], was incorporated with the sample-splitting processing in which an aliquot of peptides was dephosphorylated by phosphatase (deP). Its feasibility for determination of site-specific phosphorylation stoichiometry was demonstrated using synthetic peptides of various simulated phosphorylation degrees. Its accuracy was also validated by determining the phosphorylation degrees of milk caseins using synthetic peptide-based absolute quantification (AQUA) [21]. Moreover, this approach was applied to quantify the phosphorylation degrees of Hsp27 proteins resulting from the HepG2 cells with and without *tert*-butyl hydroperoxide (*t*-BHP) treatment. Hsp27 protein, also named the HspB1 protein, is a small heat shock protein bearing a remarkable cell protection function against oxidative stress [22]. Human Hsp27 protein contains three phosphorylatable serine sites (ser15, ser78, and ser82), which are regulated by several kinases, particularly mitogen-activated protein kinases (MAPK) and MAPK-activated protein kinases 2 and 3 (MK2,3) [23]. The change of site-specific phosphorylation of Hsp27 is proven as a crucial cellular protection mechanism, via Hsp27 oligomerization, against oxidative stress [22]. In this study, the (DM + deP) approach was further conducted to monitor the phosphorylation degree change of Ser82 on Hsp27 of HepG2 cells caused by *t*-BHP treatment.

## 2. Results and Discussion

### 2.1. Linearity and Precision of the DM + deP Approach for Quantification of the Phosphorylation Degree

**Figure 1:**
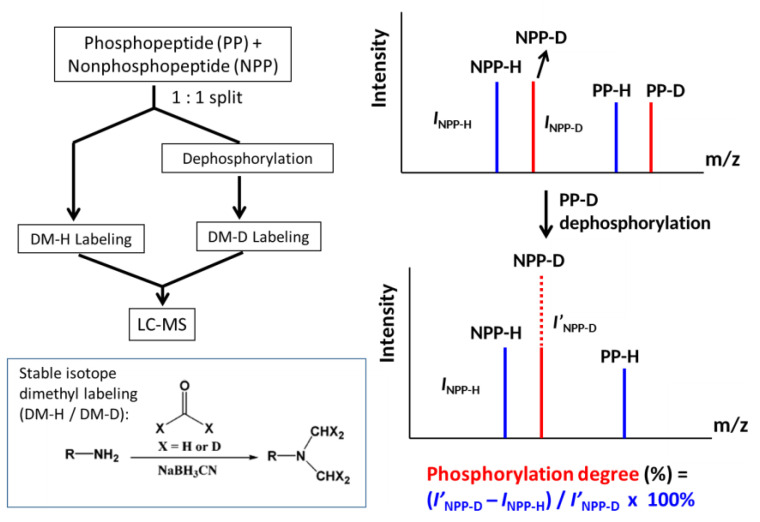
The concept and flowchart of the DM + deP approach.

The major concept of the DM + deP approach for determining the site-specific phosphorylation degrees of peptides is dependent on the quantitative increase of nonphosphorylated peptides after phosphatase dephosphorylation, as illustrated in Figure 1. For the dimethyl labeling, formaldehyde reacts with the N-terminus or ε-amino group of Lys residue of a peptide/protein in the presence of sodium cyanoborohydride (NaBH_3_CN) to form a dimethylamino group [24], as shown in Figure 1.

The peptide mixture to be assayed was divided into two aliquots simultaneously containing the phosphorylated and nonphosphorylated peptide sequences. One aliquot of the peptide mixture was directly labeled with light atom formaldehyde, while the other one was firstly dephosphorylated with phosphatase, and then labeled with a heavy atom. Afterwards, these two aliquots were combined for LC-MS analysis. In the case without phosphatase treatment, the hydrogen- and deuterium-labeled phosphorylated peptides would show the signals (*I*_PP-H_ vs. *I*_PP-D_) of equivalent intensity on the MS spectrum, and the signals of their nonphosphorylated peptides (*I*_NPP-H_ vs. *I*_NPP-D_) were also equal in intensity. After phosphatase dephosphorylation followed by heavy-atom dimethyl labeling, the increase of deuterium-labeled nonphosphorylated peptide signal (*I’*_NPP-D_) would be contributed by dephosphorylation of the phosphorylated peptides originally marked by *I*_PP-D_.

**Figure 2:**
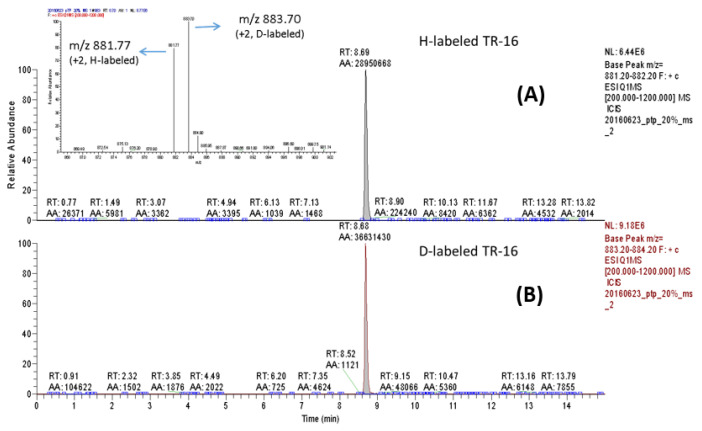
The representative LC-MS (selective ion chromatogram (SIC) of TSQ) chromatograms for quantitative analysis of site-specific phosphorylation: (**A**) the SIC of nonphosphorylated TQTPPVSPAPQPTEER (TR-16) labeled with light atom (H) dimethylation and (**B**) the SIC of D-labeled TR-16 was contributed by nonphosphorylated and dephosphorylated TQTPPVSPAPQPTEER derived from pTQTPPVSPAPQPTEER. The inserted figure shows the MS spectrum of H- and D-labeled TR-16, *m*/*z* 881.7 (+2) and 883.7 (+2), respectively.

To evaluate the compatibility and efficacy of this DM + deP method for quantification of site-specific phosphorylation, the synthetic phosphorylated peptide pTQTPPVSPAPQPTEER (pTR-16, Mw = 1812.85 Da) and its nonphosphorylated counterpart TQTPPVSPAPQPTEER (TR-16, Mw = 1732.84 Da) were used as the samples for testing. Phosphorylated and nonphosphorylated TR-16 were blended to prepare the peptide mixtures of simulated phosphorylation degrees (20%, 33%, 50%, 66%, and 80%; e.g., the peptide mixture of 20% phosphorylation was prepared by mixing 20 ng of pTR-16 and 80 ng of TR-16). Afterwards, these peptide mixtures were analyzed according to the flowchart explained in Figure 1. The selective ion chromatograms (SICs) and the MS spectrum resulting from H-labeled and D-labeled TR-16 mixtures of 20% phosphorylation are shown in Figure 2. The SIC chromatograms for the mixtures of other phosphorylation degrees are also available in Figure S1 of the Supporting Information. Based on the SIC peak areas (AA) of H-labeled TR-16 (*m*/*z* 881.77, +2) and D-labeled TR-16 (*m*/*z* 883.70, +2), the phosphorylation degrees (PD%) could be calculated through the equation: (AA’_D-labeled TR-16_ − AA_H-labeled TR-16_)/AA’_D-labeled TR-16_, similar to the operation for the MS signal intensities illustrated in Figure 1. The five peptide mixtures theoretically prepared and their experimental phosphorylation degrees obtained from the DM + deP analysis are listed in Table 1. The good linearity (R^2^ = 0.9971; Figure S2) and small standard derivation (<4%) between the theoretical and experimental values demonstrated the accuracy and precision of this method for estimating phosphorylation degrees (Table 1). In addition, there was no messy signal for remained phosphopeptide and unmodified (not dimethylated) peptide detected on the LC-MS chromatograms (Figure 2). It implies that complete dephosphorylation and dimethylation could be readily achieved when the experimental conditions described above were employed.

A similar protocol has been also adopted by some previous reports, all of which performed the phosphatase treatment before stable-isotope labeling [14,15,16]. We were curious if the processing order could be exchanged. After examination, our data (not shown) indicated that dimethyl labeling could be performed prior to phosphatase dephosphorylation, but an additional desalting step is required, because the activity of phosphatase can be affected in the medium containing formaldehyde and NaBH_3_CN.

### 2.2. Determination of Casein Peptide Phosphorylation Degrees in Milk with the DM + deP Approach

**Figure 3:**
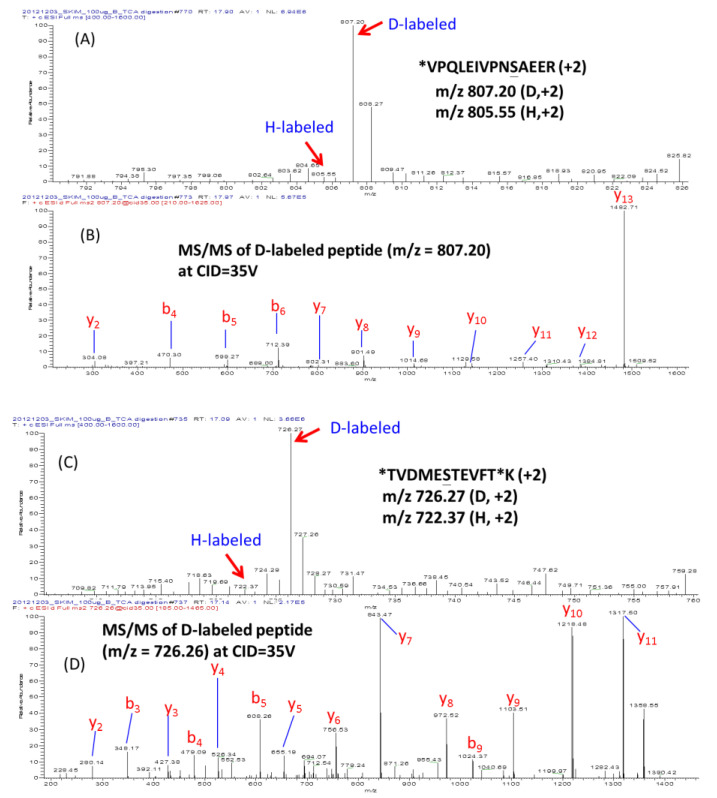
Determination of phosphorylation degrees at Ser130 of α-S1-casein and Ser158 of α-S2-casein. (**A**) LC-MS (LTQ Orbitrap) spectrum of H- and D-labeled *VPQLEIVPNSAEER, which contains Ser130 of α-S1-casein; (**B**) MS/MS spectrum of D-labeled *VPQLEIVPNSAEER; (**C**) LC-MS spectrum of H- and D-labeled *TVDMESTEVFT*K (Ser158 of α-S2-casein); and (**D**) MS/MS spectrum of D-labeled *TVDMESTEVFT*K. “*” marks the dimethyl labeling site; and “_” indicates the original phosphorylation site.

Extremely high phosphorylation degrees of bovine caseins have been reported in the literature [25,26]. Phosphorylation allows caseins to interact with calcium phosphate to form the large colloidal structure named casein micelle. Several phosphorylated peptides of milk caseins, such as VPQLEIVPNpSAEER (pVR-14, Mw = 1658.81 Da) and DIGpSEpSTEDQAMEDIK derived from α-S1-casein, TVDMEpSTEVFTK (pTK-12, Mw = 1464.62 Da) and NANEEEYSIGpSpSpSEEpSAEVATEEVK derived from α-S2-casein and ELEELNVPGEIVEpSLpSpSpSEESITR derived from β-casein, have been identified by previous studies [27,28]. Variation of phosphorylation degrees in α-caseins is especially interested, because it was revealed as one of the key factors for the technological properties of milk products [29]. The site-specific phosphorylation at Ser130 of α-S1-casein and Ser158 of α-S2-casein has been well identified [27,30]. However, the DM + deP method used in this study might not be feasible for checking phosphorylation degrees of the peptides with multiple phosphorylation sites. With the identical amino acid framework, no matter originally how many phosphorylation sites the peptides have, eventually they will produce the MS signal of the same intensity appearing on the same position of MS spectrum after complete dephosphorylation by phosphatase. Therefore this time when we applied DM + deP to analyze the α-casein peptides of milk, only the peptides with a single phosphorylation site were focused. As shown in the MS/MS spectra of Figure 3B,D, two of the single-sited phosphopeptides, pVR-14 (D-labeled pVR-14, *m*/*z* 807.20, +2), which contains Ser130 of α-S1-casein, and pTK-12 (D-labeled pTK-12, *m*/*z* 726.26, +2), which includes Ser158 of α-S2-casein, could be identified from the trypic digest of skim milk proteins. After phosphatase treatment, the quantitative information related to their phosphorylation conditions (*I*_NPP-H_ vs. *I’*_NPP-D_) could be readily obtained from their MS spectra (Figure 3A,C), and the phosphorylation degrees of pVR-14 and pTK-12 in skim milk proteins were calculated as 97.2% and 97.3%, respectively. This is the site-specific phosphorylation degree of α-caseins firstly reported at the peptide level.

**Figure 4:**
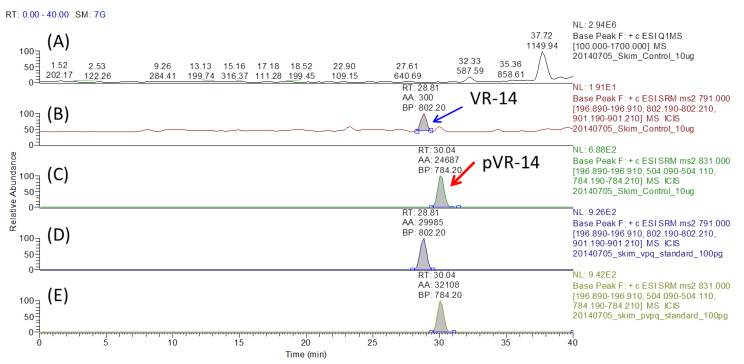
Absolute quantification (AQUA) validation of the phosphorylation degree at Ser130 of α-S1-casein with LC-MS/MS (SRM in TSQ) analysis. (**A**) The full LC-MS chromatogram for the whole tryptic digest of skim milk proteins; (**B**) the SRM chromatogram of VR-14 in tryptic skim milk digest; (**C**) the SRM chromatogram of pVR-14 in tryptic skim milk digest; (**D**) the SRM chromatogram of VR-14 (*m*/*z* 791.0 → 196.9, 802.2, 901.2) in tryptic skim milk digest added with standard VR-14 (*m*/*z* 831.0 → 196.9, 504.1, 784.2); and (**E**) the SRM chromatogram of pVR-14 in tryptic skim milk digest added with standard pVR-14.

The phosphorylation degree of Ser130 in α-S1-casein was further validated using synthetic peptide-based absolute quantification (AQUA) by LC-MS/MS analysis in the SRM mode in TSQ instrument. The full MS chromatogram for the whole tryptic digest of skim milk proteins is shown in Figure 4A. Under the collision-induced dissociation (CID) energy 42 V, three SRM transitions monitored for VR-14 (*m*/*z* 791.0, +2) were *m*/*z* 791.0 → 196.9, 802.2, and 901.2, and those for pVR-14 (*m*/*z* 831.0, +2) were *m*/*z* 831.0 → 196.9, 504.1, and 784.2, which helped determine the SRM peaks of VR-14 and pVR-14 in the tryptic digest of milk proteins, as shown in Figure 4B,C, respectively. To confirm the retention time, the synthetic VR-14 and pVR-14 were also individually mixed into the tryptic digest of milk proteins, and their corresponding SRM chromatograms are shown in Figure 4D,E, respectively. The calibration curves established from synthetic VR-14 and pVR-14 are shown in Figure S3 of the Supporting Information, and their peak areas on Figure 4B,D were converted into concentrations based on these standard curves. The phosphorylation degree of Ser130 in α-S1-casein in milk was calculated according to the equation, pVR-14 content/(pVR-14 + VR-14 contents), to give a degree of 98.9%. This absolute quantitation result was very close to that (97.2%) obtained from the DM + deP approach discussed above, which demonstrated the feasibility of the DM + deP approach in the determination of site-specific phosphorylation stoichiometry of certain (single-phosphorylated) casein peptides.

### 2.3. Relative Quantitation of Protein Phosphorylation Degree on Ser82 of Hsp27 in HepG2 Cells with and without t-BHP Treatment

**Figure 5:**
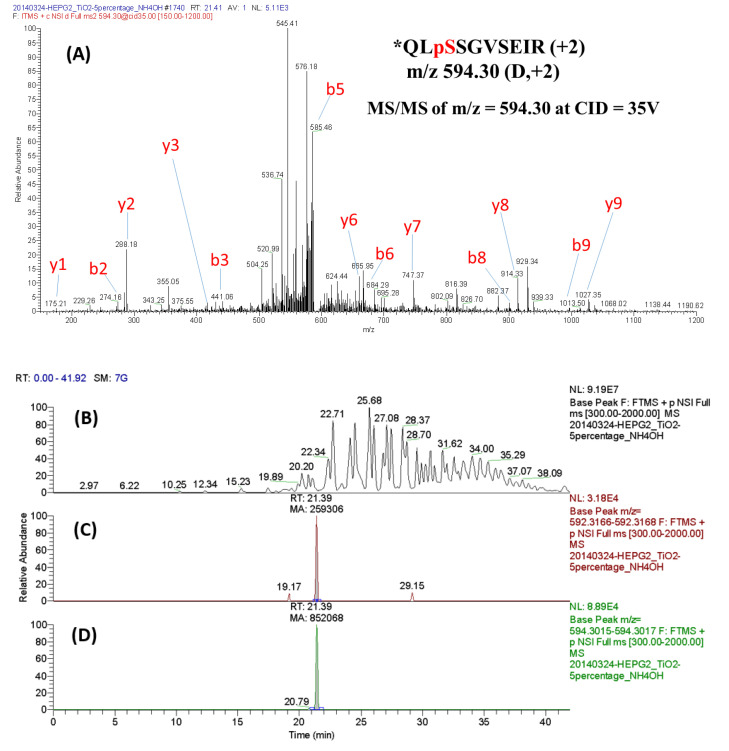
Relative quantitation of the phosphorylation degree of QLpSSGVSEIR (pQR-10) changed by oxidative treatment. (**A**) The MS/MS (LTQ Orbitrap) spectrum of D-labeled pQR-10 (*m*/*z* 594.30, +2); (**B**) the full LC-MS chromatogram of TiO_2_-enriched fraction; and (**C**) and (**D**) are the SIC of H- and D-labeled pQR-10, respectively. “*” marks the dimethyl labeling site.

The two aliquots of tryptic peptides derived from the cytosolic proteins of HepG2 cells with and without *t*-BHP treatment were labeled using formaldehyde-*d*_0_/NaBH_3_CN and formaldehyde-*d*_2_/NaBH_3_CN, respectively. The total phosphopeptides were enriched using the TiO_2_ spin tip (Titansphere Phos-TiO kit) and analyzed using LC-MS/MS coupled with Mascot database search and Mascot Distiller quantitation. Among the identified phosphorylated peptides summarized in Table S1 of the Supporting Information, D-labeled QLpSSGVSEIR (D-labeled pQR-10, *m*/*z* 594.30, 2+) with the phosphorylation site located at Ser82 of Hsp27 has been reported to be crucial for triggering Hsp27 oligomerization [22]. Therefore, we targeted at pQR-10 to explore how the phosphorylation level of Hsp27 changed under the oxidative stress caused by *t*-BHP treatment. Identification and quantitation of pQR-10 were achieved using MS/MS and LC-MS (SIC of LTQ Orbitrap) experiments, respectively, and the results are shown in Figure 5. Figure 5A displays the MS/MS spectrum of D-labeled pQR-10 (*m/z* 594.30, +2), and the full LC-MS chromatogram of the TiO_2_-enriched fraction is shown in Figure 5B. The SIC of H-labeled (derived from the control sample) and D-labeled (from the *t*-BHP treated sample) pQR-10 are shown in Figure 5C,D, respectively. The increase of the phosphorylation degree induced by the *t*-*BHP* treatment could be evaluated by the peak area ratio of D-labeled pQR-10 (*m*/*z* 594.30, +2) to H-labeled pQR-10 (*m*/*z* 592.31, +2). As shown by the calculation on Figure 5D, the phosphorylation degree of pQR-10 derived from Hsp27 could be expanded to 3.29 times by treating with 100 μM of *t*-BHP. This result is comparable to that reported by Gaitanaki et al., in which the site-specific phosphorylation at Ser82 of Hsp27 was enhanced to 5.34 fold relative to the control by treating with 30 µM H_2_O_2_ [31].

### 2.4. The Change of the Absolute Phosphorylation Degree of Hsp27 in HepG2 Cells Caused by t-BHP Treatment

**Figure 6:**
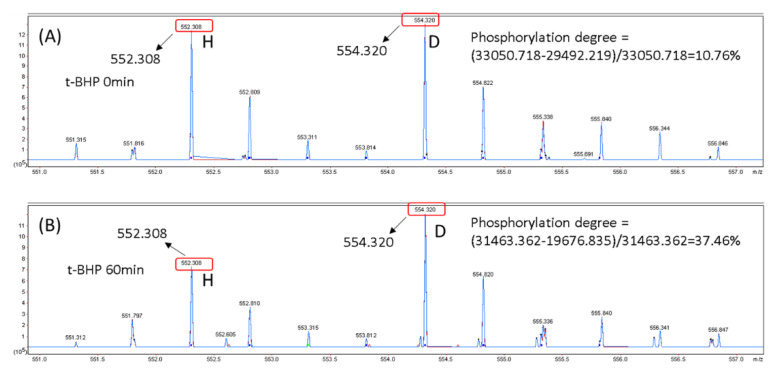
DM + deP determination of the phosphorylation degree difference at Ser82 of heat shock protein Hsp27 derived from the HepG2 cells with and without *t*-BHP treatment. (**A**) The MS spectrum (LTQ Orbitrap) of *QLSSGVSEIR (*m*/*z* 554.32 vs. *m*/*z* 552.30) without *t*-BHP treatment and (**B**) the MS spectrum (LTQ Orbitrap) of *QLSSGVSEIR after *t*-BHP treatment. “*” marks the dimethyl labeling site; “_” indicates the original phosphorylation site.

The change of the absolute phosphorylation degree of Hsp27 in HepG2 cells caused by *t*-BHP treatment was further monitored with the DM + deP approach. Without TiO_2_ enrichment, the original tryptic digest of HepG2 cell proteins was divided into two aliquots. One aliquot was directly dimethylated using the H-atom, while the other aliquot was first dephosphorylated by phosphatase and then dimethylated with the D-atom. Two aliquots were combined and analyzed using LC-MS/MS. As shown in Figure 6A, the *m*/*z* values of H-labeled QR-10 and D-labeled QR-10 from the control sample without *t*-BHP treatment were 552.308 (+2) and 554.320 (+2), respectively, and its phosphorylation degree of Ser82 on Hsp27 were determined as 10.76% by the equation “PD (%) = ((*I’_NPP-D_* − *I_NPP-H_*)/*I’_NPP-D_*) × 100%” illustrated in Figure 1. Similarly, the phosphorylation degree of Ser82 on Hsp27 with *t*-BHP treatment was determined as 37.46% via the signal intensities of H-labeled QR-10 and D-labeled QR-10 detected in Figure 6B. Based on the data resulting from the DM + deP approach, it could be inferred that the overall phosphorylation degree was increased to 3.48 (37.46%/10.76%) fold, very close to the result (3.29) obtained from relative quantitation, which were analyzed with stable-isotope dimethyl labeling coupled with TiO_2_ enrichment (Section 3.3). According to the Western blot reported by Gaitanaki et al., Hsp27 usually has a basal phosphorylation level around 10% at Ser82 in the common condition without extra exterior stress, but Ser82 of Hsp27 is sensitive to oxidative stress, and a dramatic increase of the phosphorylation degree could be induced by 30 µM H_2_O_2_ [32]. The similar does-dependent effect of H_2_O_2_ on the autophagy of rat kidney NRK-52 cells was also revealed by Matsumoto’s report in which the phosphorylation change at Ser82 of Hsp27 protein in NRK-52 cells was used as a monitor target [32].

## 3. Materials and Methods

### 3.1. Materials and Chemical Reagents

Iodoacetamide (IAM), sodium dodecyl sulfate (SDS), skim milk powder, sodium cyanoborohydride (NaBH3CN), trichloroacetic acid (TCA), *N*-octyl-β-d-glucopyranoside (NOG), *tert*-butyl hydroperoxide (*t*-BHP) solution (70% solution in H_2_O), penicillin, streptomycin, and sodium pyruvate were purchased from Sigma-Aldrich (St. Louis, MO, USA). Formic acid (FA), 1,4-dithiothreitol (DTT), ammonium bicarbonate (ABC), sodium acetate (NaOAc), ammonium hydroxide (NH_4_OH), lactic acid, and formaldehyde (37% solution in H_2_O) were purchased from J. T. Baker (Phillipsburg, NJ, USA). The shrimp alkaline phosphatase kit (#78390) was obtained from Affymetrix (Santa Clara, CA, USA). The Pierce^®^ BCA protein assay kit (#LC142892) was obtained from Thermo (Rockford, IL, USA). Sequencing grade trypsin was purchased from Promega (Madison, WI, USA). Formaldehyde-d_2_ (20% solution in D_2_O) was purchased from Aldrich (Milwaukee, WI, USA). Acetonitrile (ACN) was obtained from Merck (Darmstadt, Germany). Pyrrolidine and trifluoroacetic acid (TFA) were purchased from Alfa Aesar (Lancashire, UK). Dulbecco’s modified Eagle’s medium (DMEM) and fetal bovine serum (FBS; #10437) were purchased from GIBCO (Grand Island, NY, USA). Nucleosil RP C_18_ beads (3 μm) were obtained from Machere-Nagel (Düren, Germany). Solulyse-M lysis buffer was purchased from Genlantis (San Diego, CA, USA). Titansphere Phos-TiO kit was obtained from GL Sciences (Tokyo, Japan). HepG2 cells (BCRC 60025) were obtained from the Bioresource Collection and Research Center (Hsinchu, Taiwan). 10× phosphate-buffered saline (PBS) and synthetic peptides, including pTQTPPVSPAPQPTEER (pTR-16, Mw = 1812.85 Da), TQTPPVSPAPQPTEER (TR-16, Mw = 1732.84 Da), VPQLEIVPNpSAEER (pVR-14, Mw = 1658.81 Da), VPQLEIVPNSAEER (VR-14, Mw = 1578.81 Da), and QLpSSGVSEIR (pQR-10, Mw = 1154.55 Da), were purchased from Uni Region Bio-Tech (New Taipei City, Taiwan). The water used in this study was obtained from the Milli-Q^®^ water purification system of Millipore (Billerica, MA, USA).

### 3.2. Cell Culture and t-BHP Treatment of HepG2 Cells

HepG2 cells (BCRC 60025) were cultured in polystyrene dishes of 80.5-mm diameter (Corning, NY, USA) with DMEM containing 10% FBS, 100 μg/mL penicillin, 100 μg/mL streptomycin, and 1 mM sodium pyruvate at 37 °C under an atmosphere of 5% CO_2_. When the cell number reached 1 × 10^7^ cells per dish, the media were respectively replaced using DMEM without and with 100 μM *t*-BHP to prepare the control and *t*-BHP treated samples. After incubation for 60 min, DMEM were discarded, the cells were gently washed with PBS buffer, and then lysed with Solulyse-M lysis buffer (1 mL) for 10 min. After centrifugation (4 °C, 10,000× *g* rpm, 5 min), the supernatant was collected and the protein content was quantified using the Pierce^®^ BCA protein assay kit.

### 3.3. Trypsin Digestion of the Proteins Extracted from HepG2 Cells and Milk

Cytosolic proteins (100 μg) extracted from the HepG2 cells with and without *t*-BHP treatment were individually dissolved in deionized water (60 μL), and simultaneously, 7.5% SDS (9.3 μL) for denaturation and 1 M DTT (0.7 μL) for reduction were added to react at 95 °C for 5 min. The free Cys residues of proteins were alkylated with 0.5 M IAM (8 μL) at room temperature in the dark for 30 min. Proteins were precipitated using 50% TCA (52 μL) at ice-bath temperature. After removal of TCA and the other excess reagents, the white precipitate was dissolved in 50 mM ABC (100 μL) containing 2% NOG (5 μL), and then sequencing grade trypsin (2 μg) was added. The protein mixture was incubated at 37 °C for 18 h and the resulting peptides were acidified using 2% FA (10 μL). The whole tryptic digest was lyophilized and stored at −20 °C. Under the similar procedure, peptides were also acquired from skim milk protein (200 μg) through trypsin digestion.

### 3.4. Sample Preparation for the Relative Quantification of Phosphopeptides in the Cytosolic Proteins of HepG2 Cells with and without t-BHP Treatment

The lyophilized tryptic peptides (100 μg) derived from HepG2 cells with and without *t*-BHP treatment were individually dissolved in 100 μL of 0.1 M NaOAc, and then respectively labeled with 20% formaldehyde-d_2_ (8 μL)/1 M NaBH_3_CN (10 μL) and 37% formaldehyde (4 μL)/1 M NaBH_3_CN (10 μL), according to the previous report [24]. After being vortexed at room temperature for 30 min, the reactions were quenched with 5% NH_4_OH, then these two mixture samples were combined and desalted using a self-packed C_18_ cartridge. Afterwards, the phosphopeptides in the combined mixture were enriched using the Titansphere Phos-TiO kit according to the manufacturer’s instruction. Briefly, the spin tip (200 μL volume) was conditioned with 20 μL of buffer A (0.4% TFA in 80% can; 2000 rpm for 2 min), and then equilibrated using 20 μL of buffer B (0.3% TFA, 60% ACN, and 25% lactic acid; 2000× *g* rpm for 2 min). The combined hydrogen and deuterium-labeled peptide mixture (100 μg) in buffer B (1000 μL) was stepwise passed through the spin tip by rinsing with 20 μL of buffer B and 60 μL of buffer A at 2000× *g* rpm (2 min for every 20 μL). Finally, the phosphopeptides were eluted out from the spin tip with 10 μL of 5% NH_4_OH (1000× *g* rpm, 5 min). The resulting enriched phosphopeptides were lyophilized and dissolved in 50 μL of 0.1% FA, 20 μL of which was then subjected to a Surveyor HPLC system (Thermo Finnigan, San Jose, CA, USA) equipped with a C_18_ capillary column (3 μm bead size, 100 Å pore size, 75 μm i.d. × 15 cm, MST, Taiwan). The mobile phase, composed of solution A (0.1% FA in 5% ACN) and solution B (0.1% FA in 95% ACN), was set at a constant flow rate of 300 nL/min, and the percentage of solution B was increased from 2% to 60% in a linear gradient over 90 min. The fractionated phosphopeptides were detected using LTQ Orbitrap (Thermo Scientific, Waltham, MA, USA) equipped with nano-ESI source.

### 3.5. MS Analysis for the Relative Quantification of Phosphopeptides in the Cytosolic Proteins of HepG2 Cells with and without t-BHP Treatment

The MS scan for the precursor ion was ranged from 400 to 1600 *m*/*z*, and the MS/MS scan was processed under a data-dependent mode with a minimum count (500) for triggering MS/MS. The normalized collision energy was set at 35 V. Conversion of raw data to MGF files was performed using Mascot Distiller v2.3.2.0 (Matrix Science, London, UK), while the downstream database was searched with Mascot search engine v2.3 (Matrix Science, UK) according to the following parameters: (1) the protein database = Swiss-Prot; (2) the taxonomy = *Homo sapiens* (human; 531473 sequences); (3) the enzyme = trypsin; (4) missed cleavage allowed = 1; (5) the mass tolerance for precursor and product ion = 5 ppm/0.8 Da; (6) fixed modification = carbamidomethyl (C), dimethyl:2H(4) (K), and dimethyl:2H(4) (N-term); (7) variable modifications = oxidation (M), dimethyl (K), dimethyl (N-term), phospho (ST), and phospho (Y); and (8) the significance threshold = *p* < 0.05. For relative quantitation of the phosphorylated peptides existing in the mixture of control (H labeled) and *t*-BHP-treated (D labeled) samples, the parameters for the Mascot Distiller (Matrix Science) operation were set according to our previous report [33].

### 3.6. Absolute Quantitation of Site-Specific Phosphorylation Stoichiometry Using the DM + deP Approach

The tryptic peptides derived from the cytosolic proteins of HepG2 cells with and without *t*-BHP treatment were individually processed under the same procedure described below. The tryptic peptides (50 μg) were divided into two aliquots. One aliquot (25 μg) was directly labeled using 37% formaldehyde (4 μL)/1 M NaBH_3_CN (10 μL) in 100 μL of 0.1 M NaOAc. The other aliquot (25 μg) was dephosphorylated using the shrimp phosphatase kit (#78390) according to the manufacturer’s instruction. Briefly, 1 μL of alkaline phosphatase (0.25 U/μL) was added into the sample solution prepared by dissolving 25 μg of tryptic peptides in 50 μL of reaction buffer, and then the reaction mixture was incubated at 37 °C for 1 h. Subsequently, the enzyme was deactivated by heating at 65 °C for 15 min. The resulting dephosphorylated peptide mixture was lyophilized and dimethylated using 20% formaldehyde-d_2_ (8 μL)/1M NaBH_3_CN (10 μL) in 100 μL of 0.1 M NaOAc. The hydrogen- and deuterium-labeled aliquots were combined, desalted with a self-packed C_18_ cartridge, and then analyzed using LC-MS/MS as mentioned in Section 2.4. The change of phosphorylation degree at ser82 in Hsp27 protein caused by *t*-BHP treatment could be inferred from one of its representative peptides, QR-10 [22]. Based on the MS signal intensities of deuterium- and hydrogen-labeled QR-10 (D-labeled QR-10 and H-labeled QR-10) originated from the identical MS spectrum, the phosphorylation degree could be calculated with the equation: (D-QR-10 − H-QR-10)/D-QR-10. Via the similar method, the site-specific phosphorylation stoichiometry of skim milk tryptic peptides and synthetic milk peptide samples (pVR-14 and VR-14) could be also determined.

### 3.7. AQUA Determination of Phosphorylation Degrees of pVR-14 Derived from α-S1-Casein in Milk Using SRM

To quantify stoichiometry of the phosphorylation-sited Ser130 in α-S1-casein of milk, the synthetic pVR-14, a phosphopeptide derived from α-S1-casein [27], and its nonphosphorylated counterpart VR-14 were analyzed using LC-MS/MS in the SRM mode. Five standard solutions (5 pg/µL 10 ng/µL) for either pVR-14 or VR-14 were used to establish the standard calibration curves, which were constructed by plotting peak areas against corresponding concentrations. The slope, intercept, and correlation coefficient were obtained from least squares regression analysis. The trypsin-digested skim milk was diluted and separated using ODS Hypersil column (150 mm × 2.1 mm, 5 µm, Thermo Scientific) with the mobile phase composed of solution A (5% ACN and 0.1% FA in deionized water) and solution B (95% ACN and 0.1% FA). A linear gradient was set from 0 to 40% of solvent B over 40 min at a flow rate of 350 µL/min. The quantitative analysis was carried out under the SRM mode with a TSQ triple quadrupole mass analyzer (Quantum access max, Thermo Scientific). The ESI was performed at the positive mode with a spray voltage of 3500 V, sheath gas pressure of 40 arb, auxiliary gas pressure of 5 arb, capillary temperature of 320 °C, and tube lens offset voltage of 124 V. After obtaining the concentrations of pVR-14 and VR-14 in digested milk based on their individual standard calibration curves, the phosphorylation degree of Ser130 in α-S1-casein of milk could be calculated according to the equation: pVR-14 content/(pVR-14 + VR-14 contents).

## 4. Conclusions

Firstly, feasibility of the DM + deP approach for absolute quantitation of site-specific phosphorylation levels in proteins was proven by the samples of various phosphorylation degrees prepared from the synthetic standard phosphorylated and nonphosphorylated peptides. Afterwards, this approach was further applied to determine the phosphorylation degrees of single-phosphorylated casein peptides in milk. The site-specific phosphorylation degrees at Ser130 in α-S1-casein and Ser158 in α-S2-casein were readily determined using this DM + deP approach, and its accuracy was validated using the synthetic peptide-based absolute quantitation under SRM mode coupled with LC-MS/MS analysis. It is verified that this DM + deP method is promising for studying the formation of colloidal casein micelles, which is an important topic in dairy science. Furthermore, the DM + deP approach was also shown to be applicable to monitoring the phosphorylation degree change of Ser82 on the Hsp27 protein in HepG2 cells caused by exterior oxidative stress, which plays a crucial role in cellular protection mechanism through Hsp27 oligomerization. Our result indicated that the absolute phosphorylation degree obtained from the DM + deP approach was not only comparable with that data generated from the relative quantification method using stable-isotope dimethyl labeling coupled with TiO_2_ enrichment, but also consistent with the Western blot result reported previously. Through this study, the feasibility and accuracy of the DM + deP approach is confirmed for analyzing the site-specific phosphorylation degrees of phosphopeptides, and it is recommended to be broadly employed for the studies of cellular signal transduction.

## Figures and Tables

**Table 1:** The phosphorylation degree (PD%) was determined using the following equation: PD (%) = ((*I’_NPP-D_* − *I_NPP-H_*)/*I’_NPP-D_*) × 100%. The linearity assay of the DM + deP approach was demonstrated by the simulated phosphorylation degree using synthetic TR-16 and pTR-16.

Phosphorylation Degree (Theoretical)	Peak Area of D-Labeled Peptide	Peak Area of H-Labeled Peptide	Phosphorylation Degree (Experimental)
20%	37,032,008.5 ± 636,513	29,692,292.5 ± 600,737.4	19.82% ± 0.24%
33%	38,687,784.7 ± 3,803,881	27,327,569.7 ± 2,324,513	30.43% ± 3.75%
50%	44,018,746.7 ± 3,048,465	21,809,096 ± 1,057,211	50.38% ± 2.30%
66%	46,485,426.7 ± 4,333,081	16,914,208 ± 1,738,936	63.63% ± 0.57%
80%	53,027,741.7 ± 5,388,413	11,023,266.7 ± 318,858.1	79.07% ± 2.19%

The experiments were repeated at least three times and the results were expressed as mean ± standard deviation.

## Supplementary Materials

The following are available online. Figure S1: The selective ion chromatogram (SIC) of D-labeled and H-labeled TR-16 at different ratios. Figure S2: The linearity assay of (DM + deP) approach demonstrated using synthetic TR-16 and pTR-16. Figure S3: The SRM standard calibration curves of (A) VPQ and (B) pVPQ using synthetic peptides. Table S1: Summary of phosphopeptides identified and relatively quantified from HepG2 cells with and without *t*-BHP treatment.
