# Supplementary material for: LC-MS Quantification of Site-Specific Phosphorylation Degree by Stable-Isotope Dimethyl Labeling Coupled with Phosphatase Dephosphorylation
Source: Molecules. 2020 Nov 14;25(22):5316. doi: 10.3390/molecules25225316 (PMC7697701; doi:10.3390/molecules25225316)
Supplement: Supplementary file 1 [file molecules-25-05316-s001.pdf]

(A) The result of 20% (expected) phosphorylation of TQTPPVSPAPQPTEER (TR-16)

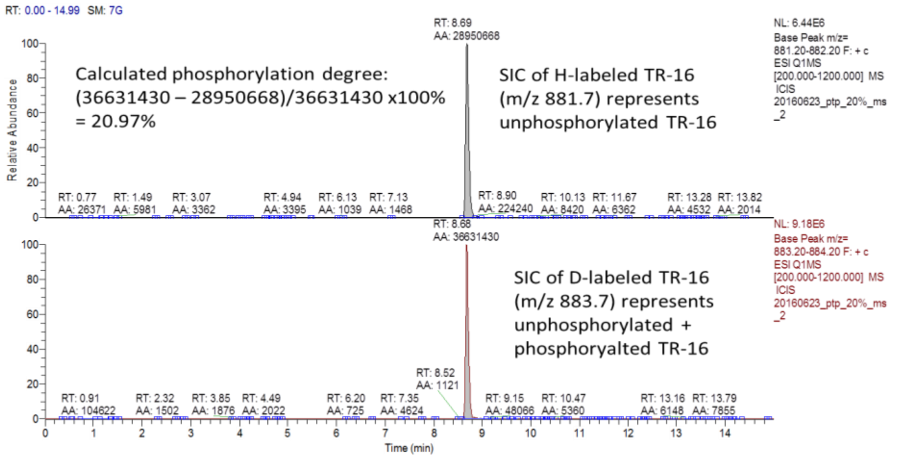

(B) The result of 33% (expected) phosphorylation of TQTPPVSPAPQPTEER (TR-16)

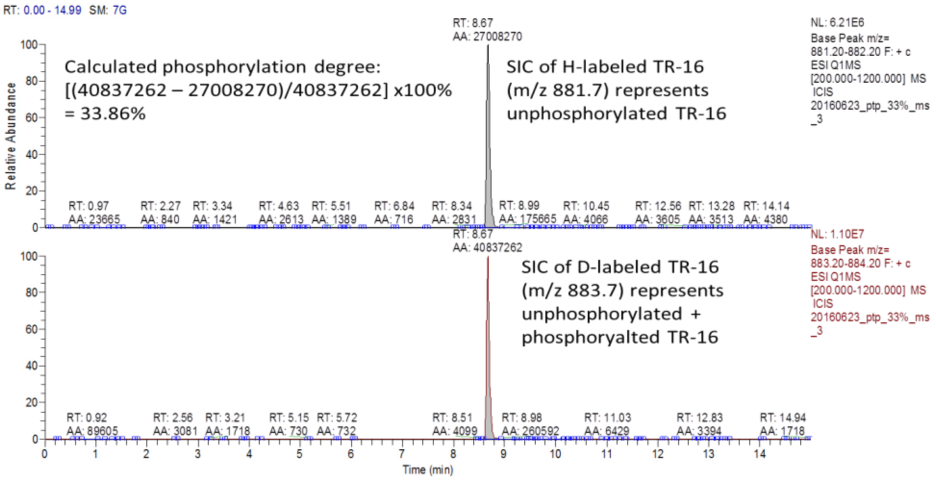

(C) The result of 50% (expected) phosphorylation of TQTPPVSPAPQPTEER (TR-16)

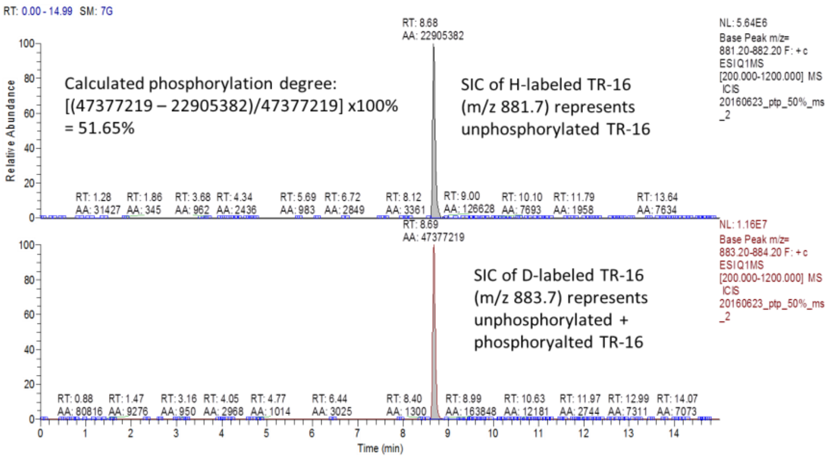

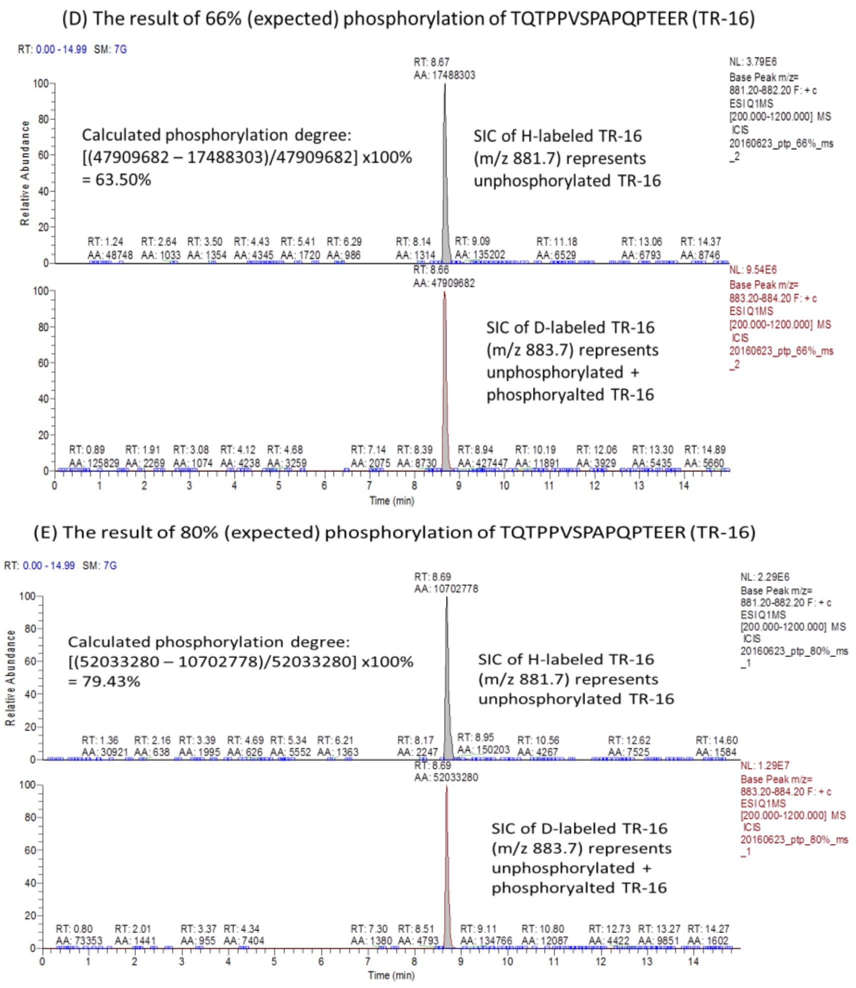

Figure S1. The selective ion chromatogram (SIC) of D-labeled and H-labeled TR-16 at different ratios. The result of (**A**) 20%, (**B**) 33%, (**C**) 50%, (**D**) 66%, and (**E**) 80% expected phosphorylation of TR-16.

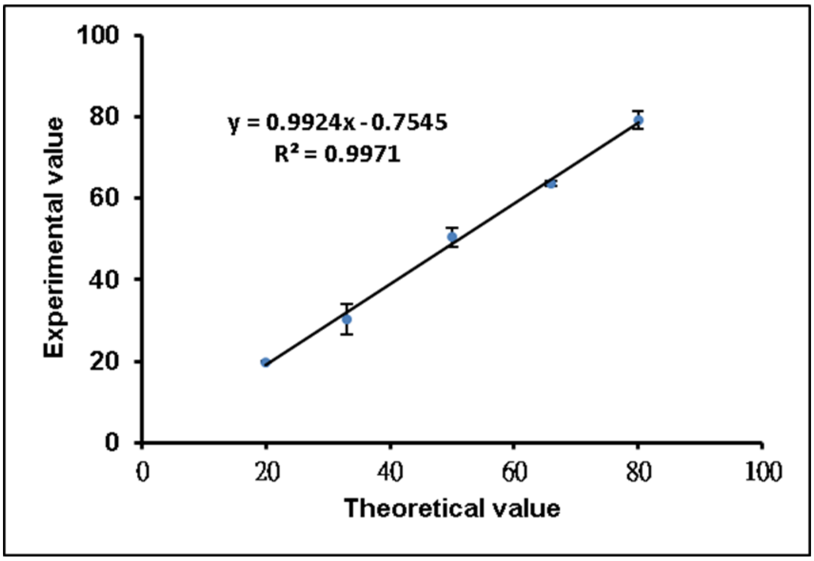

Figure S2. The linearity assay of the DM + deP approach demonstrated using synthetic TR-16 and pTR-16.

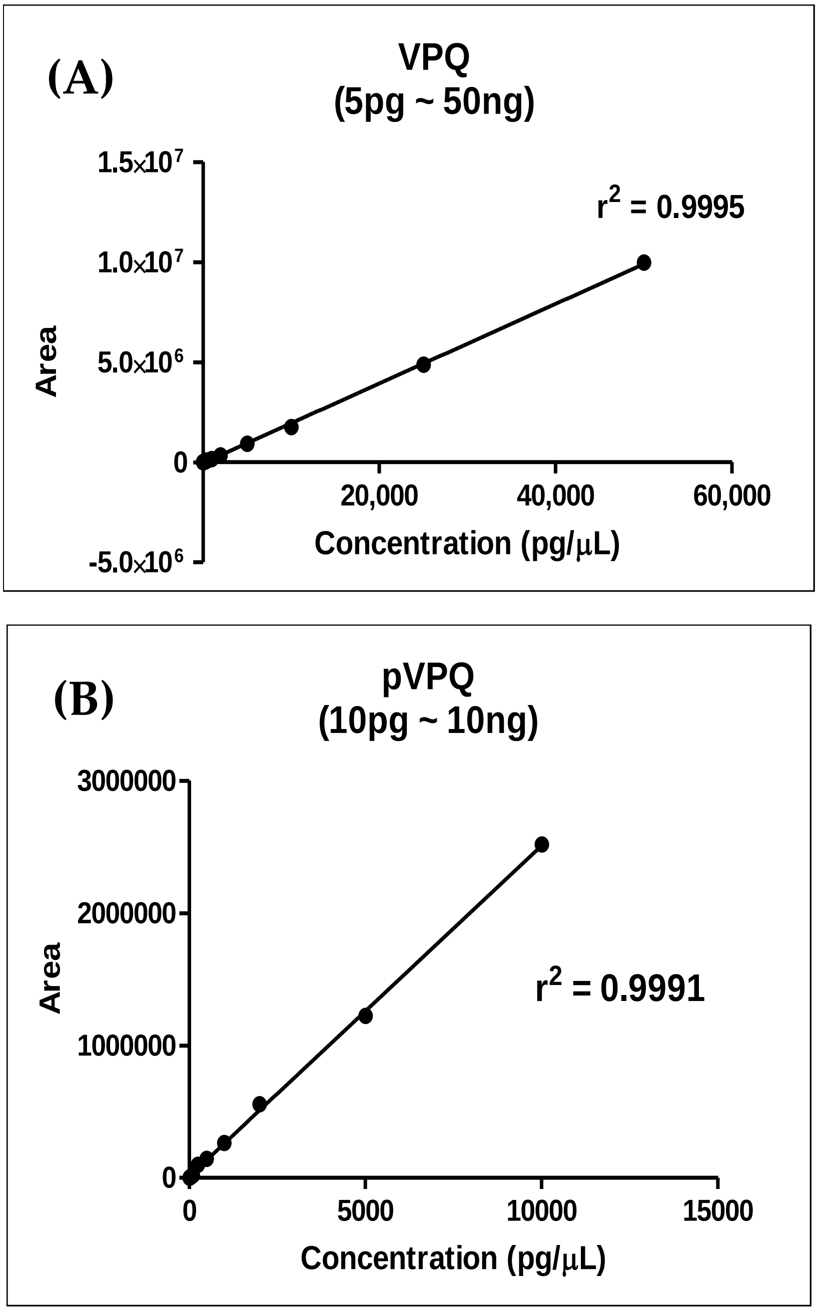

Figure S3. The SRM standard calibration curves of (A) VPQ and (B) pVPQ using synthetic peptides.

Table S1. Summary of phosphopeptides identified and relatively quantified from HepG2 cells with and without *t*-BHP treatment.

Accession No.	Peptide Sequence	Phosphopeptide Ratio (D/H)	Phosphorylation Site
IRK6_HUMAN	MAKLTESMTNVLEGDSMDQDVESPVAIHQPK	84.53	T9/S16
STMN1_HUMAN	ASGQAFELILSPR	11.94	S25
AP3D1_HUMAN	AVLIMYKVFLK	10.96	Y171
LIRB3_HUMAN	MTPALTALLCLGLSLGPRTR	5.68	T19
HSPB1_HUMAN	QLSSGVSEIR	3.29	S82
HNRPK_HUMAN	IIPTLEEGLQLPSPTATSQLPLESDAVECLNYQHYK	3.24	S116
LIRB3_HUMAN	MTPALTALLCLGLSLGPRTR	2.32	T19
RPR1B_HUMAN	TFQQIQEEEDDDYPGSYSPQDPSAGPLLTEELIK	1.29	S166
CALX_HUMAN	AEEDEILNRSPR	1.20	S583
RPR1B_HUMAN	TFQQIQEEEDDDYPGSYSPQDPSAGPLLTEELIK	1.15	S166

CALX_HUMAN	AEEDEILNRSPR	1.15	S583
RLA1_HUMAN	KEESEESDDDMGFGLFD	1.12	S104
G3BP1_HUMAN	SSSPAPADIAQTVQEDLR	1.10	S232
G3BP1_HUMAN	SSSPAPADIAQTVQEDLR	0.97	S232
XRN2_HUMAN	QAAYEMRMQNNSSPSISPNTSFTSDGSPSPLGGIK	0.70	T478
XRN2_HUMAN	QAAYEMRMQNNSSPSISPNTSFTSDGSPSPLGGIK	0.36	T478
ETFB_HUMAN	IEVIKPGDLGVDLTSK	0.06	T219
ATPO_HUMAN	LSNTQGVVSAFSTMMSVHR	Only H-form was identified	S129/T130
ETFB_HUMAN	IEVIKPGDLGVDLTSK	Only H-form was identified	T219
ATPO_HUMAN	LSNTQGVVSAFSTMMSVHR	Only H-form was identified	S129/T130
ETFB_HUMAN	IEVIKPGDLGVDLTSK	Only H-form was identified	T219
AP3D1_HUMAN	AVLIMYKVFLK	Only H-form was identified	Y171
